# Physical activity and sedentary behaviours in Greek-Cypriot children and adolescents: a cross-sectional study

**DOI:** 10.1186/1479-5868-8-90

**Published:** 2011-08-19

**Authors:** Constantinos A Loucaides, Russell Jago, Maria Theophanous

**Affiliations:** 1 Department of Education, The Open University of Cyprus, Nicosia, Cyprus; 2 Second Elementary School, Lemesos, Cyprus; 3 Centre for Exercise, Nutrition and Health Sciences, School for Policy Studies, University of Bristol, 8 Priory Road, Bristol, UK; 4 Cyprus Pedagogical Institute, Ministry of Education and Culture, Nicosia, Cyprus, 2238 Latsia, P.O. Box 12720, 2252 Nicosia, Cyprus

## Abstract

**Background:**

There are no data on physical activity and sedentary behaviours of Greek-Cypriot children and adolescents, and no study to date examined the association between these two behaviours in this population. The purpose of this study was to document the prevalence of physical activity and sedentary behaviours among Greek-Cypriot adolescents and examine the association between physical activity and a range of sedentary behaviours. Logistic regression analyses were performed to examine the association between physical activity and sedentary behaviours.

**Methods:**

A cross-sectional study among 1,966 Greek-Cypriot children and adolescents was conducted in 2008/2009. Data were collected by means of a questionnaire across primary, middle, high and technical/vocational schools.

**Results:**

Overall 52.3% and 52.4% of the participants met physical activity and television viewing guidelines respectively. Boys and younger children were more likely to meet guidelines. Boys who attended sports clubs for two or more times per week were more likely to be physically active (OR = 3.4), and those who listened to music for one or less than one hour per day were less likely to be physically active (OR = 0.6). Girls who attended sports clubs for two or more times per week and who watched television for two or less than two hours per day were more likely to be physically active, (OR = 3.0 and OR = 1.5 respectively). Girls who reported travelling by car/bus/motorbike for one or less than one hour per day were more likely to actively travel to school (OR = 1.8).

**Conclusions:**

Findings from this study provide limited support for the displacement hypothesis whereby sedentary behaviours displace physically active time. About 50.0% of Greek children and adolescents in Cyprus meet existing physical activity and television viewing guidelines. Encouraging children to attend sports clubs for at least two times per week may markedly improve their physical activity levels.

## Background

Participation in physical activity has been found to result in health benefits including improved bone mineral density and improved indices of cardiovascular health such as blood pressure and overweight and obesity among children [1-3]. Excessive television watching results in greater levels of overweight and obesity across children in many countries [4-6]. A recent study also suggested that television watching and computer use are positively associated with aggression and alcohol use [7]. Studies that examined the combined effects of television and physical activity on overweight and obesity also indicate that low levels of physical activity and high levels of television watching among younger [8] and older children [9,10] are associated with increased levels of overweight and obesity.

Current guidelines recommend that young people should engage in physical activity for at least 60 minutes of moderate to vigorous intensity per day [2,11] and should watch television for no more than 2 hours per day [12,13]. Nevertheless, findings from a number of studies from different countries suggest that young people do not meet these guidelines. Data from self-reports of children's physical activity suggest that about 42% of 12-19 year-olds from Canada [14], 35% of 15-18 years-olds from the U.S. [15] and about one-third of young people from Europe [16] meet the recommendation of at least 60 minutes of moderate to vigorous intensity activity per day. Likewise, 46% of 15-16 year-olds from Finland [17] and 45% of Scottish adolescents watch more than 2 hours of television per day [18]. A recent cross national investigation indicated that the percentage of adolescents from North America and Europe exceeding the recommended amount of television watching per day is 77% [19].

A number of studies have examined the association between physical activity and television watching. This investigation has been based on the displacement hypothesis that states that engaging in sedentary activities displaces or reduces physical activity [17,20]. A longitudinal study failed to reveal a relationship between year-to-year changes in television viewing and changes in moderate to vigorous physical activity among 10-15 year-olds suggesting that these two behaviours are two separate constructs [21]. Lack of association between television watching and other sedentary behaviours was also observed in cross-sectional studies assessing weekly frequency of physical activity participation [22], and active commuting to school [23].

On the contrary, Tammelin et al. [17] have found a negative association between television watching/computer use and self-reported physical activity in a sample of 6928, 15-16 year-old Finnish youth and Hager [24] observed in a sample of 40 boys aged 9-12 that those who watched television after school were less likely to be active as assessed by accelerometer in comparison to those who did not watch television. Reviewing the evidence on the association between sedentary behaviours and obesity development, Rey-Lopez et al. [25] concluded that it is not known whether sedentary behaviour displaces physical activity, and findings from a meta-analysis indicate that the relationship between physical activity and television watching, playing video games or using computers receives very little empirical support [26].

Findings from the above studies suggest that the evidence supporting the association between physical activity and sedentary behaviours are contradictory. It is therefore important to assess both of these behaviours as they may both need to be targeted for physical activity promotion. This is especially important as young peoples' physical activity levels tend to decline as they move through adolescence [16,27]. Furthermore, while findings regarding the association between age and screen time behaviours are mixed [6,28] a recent cross national investigation suggests that older adolescents are more likely to be spending more than two hours daily in cumulative screen time [19].

While data on physical activity among Greek-Cypriot elementary school children exist [22,29], to our knowledge there are no data that examine the physical activity levels and sedentary behaviours of Greek-Cypriot adolescents with reference to physical activity and television watching guidelines across different levels of education. This is especially important as Greek-Cypriot children's levels of overweight and obesity are increasing [30]. As obesity prevention interventions need to be tailored to the needs of local participants, an understanding of the physical activity and screen-viewing behaviours of Cypriot youth is urgently needed. Further, examining the association between physical activity and sedentary behaviours in a unique population may help enrich existing evidence. A recent study also indicated that television watching has dominated the assessment of sedentary behaviours [18], and there is a need to consider a wide range of sedentary activities when examining the association with physical activity [18,26,31]. Lastly, as only one study was located that examined the association between active commuting to school and sedentary behaviours [23], more data are needed that examine this association. To address these issues, this study examined the association between physical activity (moderate to vigorous and active travelling) and multiple sedentary behaviours in a sample of Greek-Cypriot youth.

Therefore, the purpose of this study was twofold: 1) to document the prevalence of physical activity and sedentary behaviours across different levels of education in Cyprus and 2) to examine the association between physical activity (moderate to vigorous and active travelling) and a range of sedentary behaviours.

## Methods

### Participants

Students from 25 schools from all districts under the control of the Republic of Cyprus were invited to participate in this study including grade six students from nine elementary schools (n = 448), grade 7-9 students from six middle schools (n = 656), grade 10-12 students from five high schools (n = 479) and from five technical schools (n = 383). Technical schools offer vocational rather than academic training. Letters were sent to the head-teachers of each school informing them of the procedures involved. All head-teachers gave their consent, and students from all grade six classes from the elementary schools and randomly selected classes of students from middle, high and technical schools were invited to complete questionnaires while at school. Parental informed consent was obtained by all students who completed questionnaires. The protocol for this study was approved by the Cyprus Pedagogical Institute and by the Cyprus Ministry of Education and Culture.

### Measures

#### Physical activity

Physical activity was assessed with four items modified from the Youth Risk Behavior Survey [32]. Two of these items assessed weekly frequency students participated in moderate ('Physical activity that does not make you sweat or breathe hard such as walking, slow bicycling and volleyball') and vigorous ('Physical activity that makes you sweat and breathe hard such as running, playing basketball, playing football and swimming') physical activity respectively. Responses for these items were on an eight-point scale ranging from 'not at all' to 'seven days'. Two further items assessed the usual duration that students participated in moderate and vigorous activities with four response options including 'up to 30 minutes', 'up to one hour', 'up to one and a half hour' and 'more than one and a half hour'. A recent review concluded that the Youth Risk Behavior Survey has good validity including convergent validity with accelerometry [33].

Two other items also assessed physical activity related behaviours. The first asked students to indicate their usual mode of travel to school with four possible responses including bus or car, motorcycle, bicycle and walk. The second item asked students to indicate the weekly frequency they attended a sports club. Responses for this item were on a six-point scale ranging from 'not at all' to 'more than four times'.

#### Sedentary behaviours

Eight different sedentary behaviours were assessed including television watching, video/dvd watching, playing video games (e.g. X-Box), in front of the computer, studying or doing homework, talking on the phone, listening to music, and traveling in the car/bus/motorcycle. Students were asked to indicate the usual time (hours per day) that they spent on each of the above activities. Responses were on a six-point scale and ranged from 'zero hours' to 'more than four hours'.

### Data analysis

A principal components analysis with varimax rotation was conducted on the eight items assessing sedentary activities to examine whether these sedentary behaviours could be grouped in different factors. The initial principal components analysis resulted in the extraction of two factors. However, because the internal consistency reliability of the second factor was markedly improved (from α = .56 to α = .67) after deleting the item 'hours per day studying' the factor analysis was conducted for a second time without including this item. The results of the factor analysis with the seven items included are presented in Table 1. Two factors were extracted explaining 57.70% of the variance, KMO = 0.807, Bartlett's Test of Sphericity χ^2^(21) = 2848, p < 0.001. Four items relating to screen-based activities loaded on factor one and was therefore named 'Screen-based sedentary activities' and three items loaded on factor two and was named 'Non-screen based sedentary activities'. Scores of the items that loaded on each factor were summed up and a composite score was obtained for each student on the two types of activities.

**Table 1:** Factor analysis of the items assessing sedentary behaviours

	Screen-based sedentary activities	Non-screen based sedentary activities
Hours per day watching television	.808	
Hours per day watching video/DVDs	.725	
Hours per day playing video games (e.g. X-Box, Play-station)	.714	
Hours per day spending in front of the computer	.554	

Hours per day talking on the phone		.776
Hours per day listening to music		.774
Hours per day traveling in the car/bus/motorbike		.701

Eigenvalues	2.980	1.059
Percentage of Variance explained	29.828	27.870
Cronbach's alpha for scale	.712	.674

Students were classified as physically active if they participated in moderate to vigorous physical activity for at least 60 minutes per day for seven days [2,11] and were considered to satisfy the recommendation for daily time watching television if they watched two or less than two hours of television per day [12,13]. Independent samples t-tests were employed to examine potential differences between boys and girls and physically active and inactive students across the eight sedentary behaviours and the weekly frequency of sports club attendance. Effect sizes (Cohen's d) were also calculated to examine the practical significance of the differences between group means [34]. Chi-square tests were used to examine potential differences in the percentages of adolescents across gender and level of education that satisfied the physical activity and television viewing recommendations.

Three series of adjusted logistic regression analyses (one for each of the genders and one for the whole sample) were performed with physical activity (60 or more minutes of moderate to vigorous physical activity per day versus less activity) as the dependent variable and each of the sedentary behaviours and the variable assessing weekly frequency of sports club attendance as the independent variables. The same sets of analyses were repeated with travel mode status (active versus non-active traveling to school) as the dependent variable. Students were classified as active travelers if they reported as usual mode of travel to school walk or bicycle. Reponses on time spent in sedentary behaviours and weekly frequency of sports clubs attendance were dichotomized based on median values. Independent variables with a significant association with the dependent variable at the bivariate level were entered in a logistic regression model. Level of entry at the model was set at p = 0.01. Because students were nested in schools, we used robust (Huber-White sandwich estimates) standard errors to take account of clustering (non-independence between pupils from the same school) in the computation of 95% confidence intervals and p-values. Analyses were performed using the Complex Samples procedure in the Statistical Package for the Social Science (PASW Statistics 18.0, Chicago, IL, USA) and alpha was set at 0.05.

## Results

Out of the 1966 students who completed questionnaires, 52.4% were boys. Mean age of participants was 14.7 ± 2.2. The majority of participants (84.2%) lived in the four towns of Cyprus (Nicosia, Lemesos, Larnaca, Paphos) and the rest lived in rural areas. Tables 2 and 3 present the results of the independent samples t-tests across gender (boys and girls), and physical activity (active and inactive) respectively, on each of the sedentary activity items, the two composite sedentary activity variables and the item assessing times per week attending sports clubs. The only large effect size difference between boys and girls was observed on the item assessing hours per day playing video games, whereas the only large effect size difference between active and inactive children was observed on the item assessing times per week attending sports clubs.

**Table 2:** Descriptive statistics and t-tests of gender differences in sport club attendance and sedentary activities

	Gender							Whole sample	
	Boys		Girls						
	Mean	SD	Mean	SD	t-value (df)	p-value	Effect size ^a^	Mean	SD
Times per week attending sports clubs	2.2	1.8	1.5	1.6	9.54 (1894.3)	< 0.001	0.4	1.9	1.8
Hours per day watching television	2.6	1.4	2.8	1.4	2.96 (1916)	< 0.01	0.1	2.7	1.4
Hours per day watching video/DVDs	1.7	1.4	1.4	1.3	4.72 (1900.3)	< 0.001	0.3	1.6	1.4
Hours per day playing video games (e.g. X-Box, Play-station)	2.1	1.7	0.8	1.3	18.34 (1836.4)	< 0.001	0.9	1.5	1.6
Hours per day spending in front of the computer	2.1	1.7	2.0	1.6	-	n.s.	-	2.1	1.6
Hours per day doing homework	1.5	1.3	1.9	1.2	7.74 (1894.0)	< 0.001	0.3	1.7	1.3
Hours per day talking on the phone	1.3	1.5	1.7	1.5	5.02 (1897)	< 0.001	0.3	1.5	1.5
Hours per day listening to music	2.3	1.7	2.6	1.6	4.00 (1894.0)	< 0.001	0.2	2.5	1.7
Hours per day traveling in the car/bus/motorbike	1.6	1.6	1.5	1.4	-	n.s	-	1.5	1.5
Hours per day spent on screen-based sedentary activities	8.5	4.8	7.1	4.0	6.88 (1825.7)	< 0.001	0.3	7.7	4.5
Hours per day spent on non-screen based sedentary activities	5.2	3.8	5.8	3.5	3.41 (1856.2)	< 0.01	0.2	5.4	3.6

^a ^Effect sizes of the significant differences. Values of 0.2, 0.5, and 0.8 are interpreted as small, medium, and large effect sizes, respectively.

**Table 3:** Descriptive statistics and t-tests of differences between inactive and active students on sport club attendance and sedentary activities

	Physical Activity						
	Inactive		Active				
	Mean	SD	Mean	SD	t-value (df)	p-value	Effect size ^a^
Times per week attending sports clubs	1.1	1.4	2.5	1.8	18.26 (1811.5)	< 0.001	0.9
Hours per day watching television	2.7	1.5	2.6	1.4	2.70 (1833.4)	< 0.01	0.1
Hours per day watching video/DVDs	1.6	1.4	1.6	1.3	-	n.s	-
Hours per day playing video games (e.g. X-Box, Play-station)	1.4	1.6	1.6	1.6	2.81 (1847.0)	< 0.01	0.1
Hours per day spending in front of the computer	2.1	1.7	2.0	1.6	-	n.s.	-
Hours per day doing homework	1.7	1.3	1.7	1.3	-	n.s	-
Hours per day talking on the phone	1.6	1.5	1.4	1.5	3.18 (1831.38)	< 0.01	0.1
Hours per day listening to music	2.4	1.7	2.5	1.7	-	n.s	-
Hours per day traveling in the car/bus/motorbike	1.5	1.4	1.5	1.5	-	n.s	-
Hours per day spent on screen-based sedentary activities	7.8	4.4	7.7	4.4	-	n.s	-
Hours per day spent on non-screen based sedentary activities	5.5	3.6	5.3	3.6	-	n.s	-

^a ^Effect sizes of the significant differences. Values of 0.2, 0.5, and 0.8 are interpreted as small, medium, and large effect sizes, respectively.

### Physical activity and television viewing prevalence

**Figure 1:**
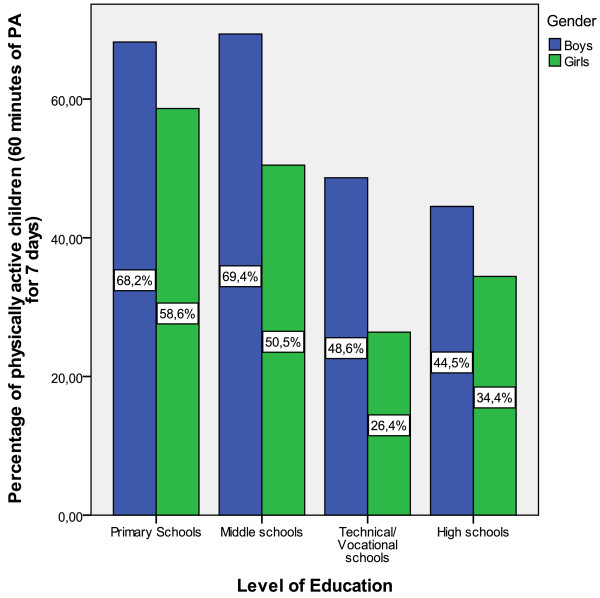
**Percentages of adolescents across gender and level of education that meet physical activity recommendations**. Statistically significant gender differences in primary (p < 0.05), middle (p < 0.001), technical (p < 0.01) and secondary schools (p < 0.05). Statistically significant differences between primary and technical (p < 0.001) and between primary and secondary schools (p < 0.001) and statistically significant differences between middle and technical (p < 0.001) and between middle and secondary schools (p < 0.001).

Overall 52.3% of the participants were classified as physically active, with boys more likely to be physically active than girls, (χ^2^(1) = 36.19, p < 0.001) (59.0% versus 45.2%). Statistically significant gender differences were observed across all levels of education. A statistically significant difference was also observed across levels of education, (χ^2^(3) = 83.33, p < 0.001) with a higher percentage of students from primary and middle schools meeting physical activity recommendations in comparison to students from technical and secondary schools. Figure 1 presents the percentages of physically active students across gender and level of education.

**Figure 2:**
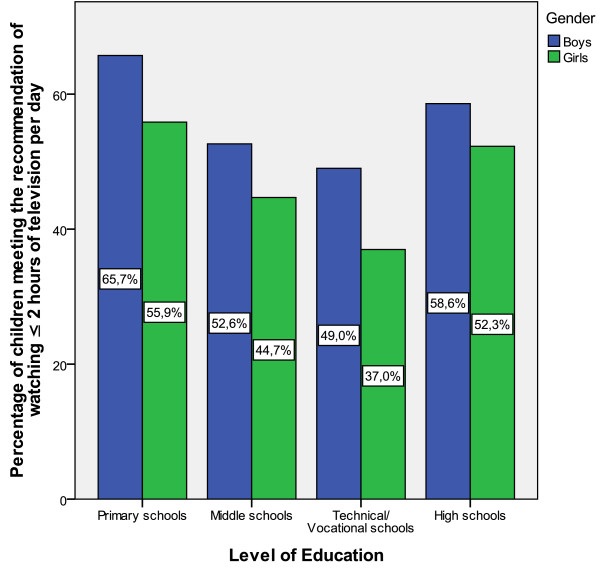
**Percentages of adolescents across gender and level of education that meet television viewing recommendation**. Statistically significant gender differences in primary (p < 0.05) and middle (p < 0.05) schools. Statistically significant differences between primary and middle (p < 0.001) and between primary and technical/vocational schools (p < 0.001) and statistically significant differences between high and technical schools (p < 0.05).

On the whole, 52.4% of the participants met the recommendation of watching ≤ 2 hours of television per day with boys more likely to meet the recommendation, (χ^2^(1) = 6.87, p < 0.01) (55.3% versus 49.3%). Statistically significant gender differences were observed across primary and middle schools. A statistically significant difference was also observed across levels of education, (χ^2^(3) = 23.31, p < 0.001) with a higher percentage of students from primary schools meeting recommendations in comparison to students from middle and technical schools and a higher percentage of students from high schools meeting recommendations in comparison to students from technical schools. Figure 2 presents the percentages of students meeting the recommendation across gender and level of education.

### Associations between physical activity and sedentary behaviours

Table 4 presents the results of the logistic regression analyses with physical activity level (inactive versus active) as the dependent variable. Boys from high schools and technical education schools were less likely to be physically active (OR = 0.4, 95%CI: 0.3-0.6 and OR = 0.5, 95%CI: 0.3-0.8 respectively) than boys from primary schools. Boys who attended sports clubs for two or more times per week were more likely to be physically active, (OR = 3.4, 95% CI: 2.7-4.2), and boys who listened to music for one or less than one hour per day were less likely to be physically active, (OR = 0.6, 95% CI: 0.5-0.8). Girls from high schools and technical education schools were less likely to be physically active (OR = 0.5, 95%CI: 0.3-0.8 and OR = 0.4, 95%CI: 0.2-0.8 respectively) than girls from primary schools. Girls who attended sports clubs for two or more times per week were more likely to be physically active, (OR = 3.0, 95% CI: 2.2-4.2), and girls who watched television for two or less than two hours per day were more likely to be physically active, (OR = 1.5, 95% CI: 1.1-2.1).

**Table 4:** Logistic regression models predicting activity status (non-active versus active) from sports club attendance and sedentary activities

	Boys (N = 937)				Girls (N = 891)				Whole sample (N = 1818)			
	Unadjusted		Adjusted		Unadjusted		Adjusted		Unadjusted		Adjusted	
	OR^a^	95%CI^b^	OR	95%CI	OR	95%CI	OR	95%CI	OR	95%CI	OR	95%CI
**Level of Education**												
Primary	Ref.		Ref.		Ref.		Ref.		Ref.		Ref.	
Middle	1.1	0.6-1.8	1.0	0.6-1.8	0.7	0.5-1.1	0.9	0.6-1.3	0.9	0.6-1.3	1.0	0.7-1.5
High	0.4	0.2-0.6***	0.4	0.3-0.6 ***	0.4	0.2-0.6***	0.5	0.3-0.8**	0.4	0.3-0.5***	0.5	0.3-0.7***
Technical	0.4	0.3-0.7 ***	0.5	0.3-0.8 **	0.3	0.1-0.5 ***	0.4	0.2-0.8**	0.5	0.3-0.7 **	0.6	0.4-0.9 *
**Sports clubs**												
< 2 times	Ref.		Ref.		Ref.		Ref.		Ref.		Ref.	
≥ 2 times	3.9	3.1-4.9 ***	3.4	2.7-4.2 ***	3.6	2.5-5.1 ***	3.0	2.2-4.2 ***	3.9	3.1-5.0 ***	3.4	2.7-4.4 ***
**Television**												
> 2 hours	Ref.		-		Ref.		Ref.		Ref.		Ref.	
≤ 2 hours	0.9	0.7-1.2			1.6	1.2-2.1 **	1.5	1.1-2.1 **	1.2	1.0-1.5	1.2	0.9-1.5
**Video/DVDs**												
> 1 hour	Ref.		-		Ref.		-		Ref.		-	
≤ 1 hours	1.0	0.8-1.3			1.0	0.7-1.5			1.0	0.8-1.2		
**Electronic games**												
> 1 hour	Ref.		-		Ref.		-		Ref.		Ref.	
≤ 1 hours	1.0	0.8-1.2			0.9	0.6-1.2			0.8	0.6-0.9 *	0.9	0.7-1.1
**Computer**												
> 2 hours	Ref.		-		Ref.		-		Ref.		-	
≤ 2 hours	0.9	0.6-1.2			1.2	0.9-1.7			1.0	0.8-1.3		
**Homework**												
> 1 hour	Ref.		-		Ref.		-		Ref.		-	
≤ 1 hours	0.7	0.5-1.1			0.9	0.6-1.3			0.9	0.7-1.2		
**Talking on the phone**												
> 1 hour	Ref.		-		Ref.		Ref.		Ref.		Ref.	
≤ 1 hours	1.2	0.8-1.7			1.4	1.0-2.0 *	1.1	0.8-1.6	1.4	1.0-1.8 *	1.1	0.8-1.5
**Listening to Music**												
> 1 hour	Ref.		Ref.		Ref.		-		Ref.		-	
≤ 1 hours	0.8	0.6-1.0	0.6	0.5-0.8 **	1.0	0.7-1.3			0.9	0.7-1.1		
**Traveling by car/bus**												
> 1 hour	Ref.		-		Ref.		-		Ref.		-	
≤ 1 hours	1.3	0.8-1.9			1.2	0.8-1.7			1.1	0.8-1.4		
**Sum of screen based**												
> 7 hours	Ref.		-		Ref.		-		Ref.		-	
≤7 hours	1.0	0.8-1.2			1.2	0.8-1.8			1.0	0.8-1.3		
**Sum of non screen based**												
> 5 hours	Ref.		-		Ref.		-		Ref.		-	
≤5 hours	0.9	0.6-1.2			1.2	0.9-1.7			1.1	0.8-1.4		

^a^OR = Odds Ratio, ^b^CI = Confidence Interval; Note: Entry level to the logistic regression model was set at p < 0.10; * association at p < 0.05; ** association at p < 0.01; *** association at p < 0.001.

Additional File 1 presents the results of the logistic regression analyses with travel mode status to school (inactive versus active travel) as the dependent variable. Boys from technical education schools were less likely to travel by active mode to school (OR = 0.3, 95% CI: 0.2-0.5) than boys from primary schools. Girls who reported travelling by car/bus/motorbike for one or less than one hour per day were more likely to actively travel to school, (OR = 1.8, 95% CI: 1.1-2.9).

## Discussion

The purpose of this study was to examine the prevalence of physical activity and sedentary behaviours in a sample of Greek children and adolescents in Cyprus and present evidence on the association between these two behaviours. On the whole, 52.3% of the participants met current guidelines recommending that young people should engage in moderate to vigorous physical activity for at least 60 minutes per day. These prevalence estimates are slightly higher than self-reported estimates reported in the US and Canada [14,15]. While, in general, about one third of young people from European countries meet these recommendations, according to Armstrong and Welsman [16] comparison between countries should be made with caution as wide variations are observed across countries. Further, comparison is even more complicated because of the different measures adapted in each study to measure physical activity. For example, studies using accelerometers in national and international studies, indicate that the proportion of adolescents meeting these recommendations vary between 2.0 to 61.0% [35] and 62.0% to 97.6% [27]. As this is the first study that presents data on physical activity prevalence based on international guidelines among Cypriot youth from different ages, it may be used for comparison purposes until more data from a more representative sample using objective measures of physical activity is obtained.

Our results indicate that boys are more active than girls across all levels of education with the highest prevalence estimates observed among boys from middle and primary schools (69.4% and 68.2% respectively) and the lowest among girls from technical and high schools (26.4% and 34.4% respectively). Further, their appears to be a marked decline in children's physical activity levels after middle school (i.e. after 14-15 years of age) whereby the overall percentages of physical active adolescents in primary and middle schools were 63.2% and 59.8% respectively while the respective percentages for high and technical schools were 37.8% and 44.3%. These findings are in agreement with studies from European countries [16,27] and from North America [14,35] indicating gender and age related differences in physical activity levels. Interestingly, in the study by Whitt-Glover et al. [35] age-related differences were observed from the age of 12, while in the current study the marked decrease was observed in the age of 15. This may be partly explained by the increased homework obligations among older students. These findings suggest that girls may be especially targeted for physical activity interventions as well as children older than 15 in order to reduce the marked decline of physical activity observed.

Sedentary activities that children devoted most of their time to included television watching (2.7 hours per day), listening to music (2.5 hours per day), in front of the computer (2.1 hours per day) and doing homework (1.7 hours per day). These findings are similar to studies from Scotland [18] and Hungary [36] where television watching, doing homework, and playing computer/video games were among the top five most time consuming sedentary activities. The only large effect size difference observed between boys and girls was in hours per day playing video games (means were 2.1 and 0.8 respectively), a finding that confirms findings from other countries [37,38]. Further, mean hours per day spent watching television in the current study are within the range (1.8 to 2.8 hours per day) reported in a review study by Marshall et al. [37]. Total daily hours spent on screen-based activities (television, video games, DVDs, computer) and non screen-based sedentary activities (talking on the phone, listening to music and motorized transport) were 7.7 and 5.4 respectively. While time spent in both of these types of sedentary behaviours appears to be extensive, it should be noted that screen based activities may be done concurrently with non-screen based activities such as watching television and listening to music or talking on the phone. Interestingly, both of these values observed in the current study are in the range of 5.5 to 8.5 accelerometer derived mean hours per day spent in sedentary activities reported by Whitt-Glover et al. [35] in a large sample of adolescents from the US.

The finding that about half (52.4%) of the adolescents met the recommendation of watching television for less than two hours per day indicates that there is a need to reduce the time spent in front of the television. These estimates are comparable to data from 11-15 year-old children from mainland Greece [19] and 15-16 year-old Finnish adolescents [17] but are more favorable than estimates from children in Italy [38] and Canada [39] where 38.0% and 25.0% of children respectively met the recommendation of watching television for less than two hours per day. In our study, boys were more likely to meet the recommendation in comparison to girls (55.3% versus 49.3% respectively), a finding that contradicts findings from previous studies [17,19,39]. This finding may be partly explained by the large amount of time that boys spent in other competing sedentary activities such as video game playing, or other pursuits such as sports clubs attendance as observed in this study. Boys from primary and high schools were most likely to meet recommendations (65.7% and 58.6% respectively) and girls from technical and middle schools were the least likely to meet recommendations (37.0% and 44.7% respectively). In general, our findings support previous work that suggests that the percentage of children that meets the recommendations decreases as they grow older [38,39]. Of interest is the low percentage of girls from technical and middle schools that meet recommendations, a finding that suggests that these groups should be especially targeted for intervention programmes.

In general, our findings provide limited support for the displacement hypothesis, as only two significant associations in the subgroup analyses were observed between physical activity and sedentary behaviours. Interestingly, boys who listened to music for less than one hour per day were less likely to be active in comparison to those who listened to music for more than one hour per day. To our knowledge, this is a novel finding and more research is needed to confirm the present association. A possible explanation may be that during physical activity young people may find music both enjoyable and motivating [40] and therefore, those who listen to music for more than one hour per day may simultaneously be more likely to engage in physical activity. The only significant association observed between physical activity and screen based activities was in the girls' analyses where those girls who watched television for less than two hours per day were more likely to be physically active. Previous research has produced contrasting results with some studies failing to show any associations [21,22], other studies showing associations only with boys [24] and other studies showing small associations with the whole sample [17].

While our study assessed the association between physical activity and a number of sedentary behaviours as well as between physical activity and composite variables of screen-based and non-screen based sedentary activities, the fact that only two significant associations were observed supports previous research that physical and sedentary behaviours are two separate constructs [21] and that both need to be targeted in potential intervention programmes to promote physical activity. This is also enhanced by the lack of a significant association between active commuting to school and screen-based sedentary activities, a finding that supports a previous study conducted in Canada [23].

Another important finding of the present study is the strong association between physical activity and weekly times of sports clubs attendance whereby children who attended sports clubs for two or more times per week were at least three times more likely to meet physical activity recommendations. This finding supports previous results with Greek-Cypriot children using a four-day physical activity recall [41] and pedometers [42] as well as findings from the United States using accelerometers, where children accumulated additional 20-minutes of moderate-to-vigorous-activity while attending after school programmes [43]. A higher percentage of boys than girls (61.6% and 44.6% respectively) reported attending sports clubs for two or more times per week. Furthermore, there was a graded decrease in the percentages of adolescents attending sports clubs for two or more times per week from primary (68.8%), middle (58.6%), technical and high schools (45.7% and 38.5% respectively). These differences in sports clubs attendance may partly explain gender and age related differences in the percentages of adolescents meeting physical activity recommendations.

While, to our knowledge, this is the first study to examine physical activity and sedentary behaviours in relation to appropriate guidelines in a large sample of Greek children and adolescents in Cyprus from different levels of education, a number of limitations are also worth addressing. First, the cross-sectional design of the present study precludes the inference of cause and effect relationships between physical activity and sedentary behaviours. Second, physical activity was assessed via self-report and future studies within the Cypriot context that examine the relationship between physical activity and sedentary behaviours should adopt objective measures of physical activity including accelerometers or pedometers. Incorporating an objective measure of physical activity behaviour, at least from a subsample, would strengthen the results of this study. Third, while a number of sedentary activities were assessed, students were asked to indicate the usual time (hours per day) that they spent on each of the above activities. Reporting sedentary activities using one-day recalls or diaries, rather than using 'the usual time' might have improved the validity of these measures. While assessing sedentary behaviours such as television viewing with single items is subject to measurement error, this approach has been used in a large number of studies and is appropriate for surveillance studies [44]. Fourth, socioeconomic status data were not collected in this study and possible differences in SES between levels of education might have biased the results. Furthermore, assessment of physical activity and sedentary behaviours did not differentiate between weekdays and weekends.

## Conclusions

Our results indicate that about 50.0% of Greek children and adolescents in Cyprus meet existing physical activity and television viewing guidelines with marked gender and educational level differences. This study provided limited support for the displacement hypothesis indicating that both physical activity and sedentary behaviours need to be targeted when implementing intervention programmes for promoting physical activity and decreasing sedentary behaviours. Encouraging children to enroll and attend sports clubs for at least two times per week may markedly improve their physical activity levels.

## Competing interests

The authors declare that they have no competing interests.

## Authors' contributions

CAL and MT designed the study and collected the data. CAL, MT and RJ conceived the paper. RJ helped plan the statistical analyses and CAL completed data analyses. CAL and MT drafted the manuscript and RJ critically revised subsequent versions of the paper. All authors read and approved the final version of the paper.

## Acknowledgements

Special thanks are due to the children who participated in this study, to the teachers who devoted their valuable time for data collection and to the two Inspectors of Home Economics of the Cyprus Ministry of Education, Ms Eva Neophytou and Ms Sandry Taliadorou.

This report is also research arising from a Career Development Fellowship (to Dr Jago) supported by the National Institute for Health Research. The views expressed in this publication are those of the authors and not necessarily those of the NHS, the National Institute for Health Research or the Department of Health.

## Supplementary Material

Additional file 1 **Logistic regression models predicting travel mode to school (non-active versus active traveling) from sports club attendance and sedentary activities**. This table presents odds ratios and confidence intervals from the analyses examining the association between travel mode to school (non-active versus active traveling), sports club attendance and sedentary activities. Click here for file
