# Supplementary material for: Physical activity and sedentary behaviours in Greek-Cypriot children and adolescents: a cross-sectional study
Source: Int J Behav Nutr Phys Act. 2011 Aug 19;8:90. doi: 10.1186/1479-5868-8-90 (PMC3176145; doi:10.1186/1479-5868-8-90)
Supplement: Additional file 1 — Logistic regression models predicting travel mode to school (non-active versus active traveling) from sports club attendance and sedentary activities. This table presents odds ratios and confidence intervals from the analyses examining the association between travel mode to school (non-active versus active traveling), sports club attendance and sedentary activities. [file 1479-5868-8-90-S1.DOC]

Additional File 1: Logistic regression models predicting travel mode to school (non-active versus active traveling) from sports club attendance and sedentary activities.

|  | Boys (N = 982) | | | | Girls(N = 897) | | | | Whole sample (N = 1839) | | | |
| --- | --- | --- | --- | --- | --- | --- | --- | --- | --- | --- | --- | --- |
|  | Unadjusted | | Adjusted | | Unadjusted | | Adjusted | | Unadjusted | | Adjusted | |
|  | OR^a^ | 95%CI^b^ | OR | 95%CI | OR | 95%CI | OR | 95%CI | OR | 95%CI | OR | 95%CI |
| **Level of Education**  Primary  Middle  High  Technical | Ref.  0.7  0.9  0.3 | 0.4-1.4  0.5-1.6  0.2-0.5^***^ | Ref.  0.7  0.9  0.3 | 0.4-1.4  0.5-1.6  0.2-0.5^***^ | Ref.  1.2  1.0  0.4 | 0.6-2.1 0.5-1.8 0.2-1.0^*^ | Ref.  1.3  1.2  0.5 | 0.7-2.4 0.6-2.3 0.2-1.3 | Ref.  0.9  0.9  0.4 | 0.6-1.5 0.5-1.5 0.3-0.6^***^ | Ref.  1.0  1.0  0.5 | 0.6-1.6 0.6-1.6 0.3-0.7^**^ |
| **Sports clubs**  <2 times  ≥ 2 times | Ref.  1.3 | 0.9-1.8 | - |  | Ref.  1.1 | 0.7-1.6 | - |  | Ref.  1.2 | 0.9-1.5 | - |  |
| **Television**  >2 hours  ≤ 2 hours | Ref.  1.3 | 0.9-1.8 | - |  | Ref.  1.1 | 0.8-1.6 | - |  | Ref.  1.2 | 0.9-1.6 | - |  |
| **Video/DVDs**  >1 hour  ≤ 1 hours | Ref.  1.2 | 0.9-1.7 | - |  | Ref.  1.0 | 0.6-1.6 | - |  | Ref.  1.1 | 0.8-1.4 | - |  |
| **Electronic games**  >1 hour  ≤ 1 hours | Ref.  1.1 | 0.8-1.5 | - |  | Ref.  0.9 | 0.6-1.4 | - |  | Ref.  1.0 | 0.8-1.3 | - |  |
| **Computer**  >2 hours  ≤ 2 hours | Ref.  1.1 | 0.8-1.6 | - |  | Ref.  1.2 | 0.9-1.6 | - |  | Ref.  1.2 | 0.9-1.5 | - |  |
| **Homework**  >1 hour  ≤ 1 hours | Ref.  0.8 | 0.6-1.1 | - |  | Ref.  0.9 | 0.7-1.3 | - |  | Ref.  0.9 | 0.7-1.1 | - |  |
| **Talking on the phone**  >1 hour  ≤ 1 hours | Ref.  1.3 | 0.9-2.0 | - |  | Ref.  1.3 | 0.9-1.9 | Ref.  1.0 | 0.7-1.4 | Ref.  1.3 | 1.0-1.8 | Ref.  1.0 | 0.7-1.4 |
| **Listening to Music**  >1 hour  ≤ 1 hours | Ref.  1.3 | 0.9-2.0 | - |  | Ref.  0.9 | 0.7-1.1 | - |  | Ref.  1.1 | 0.8-1.4 | - |  |
| **Traveling by car/bus**  >1 hour  ≤ 1 hours | Ref.  1.3 | 0.8-1.9 | - |  | Ref.  2.1 | (1.4-3.1)^**^ | Ref.  1.8 | 1.1-2.9^*^ | Ref.  1.6 | 1.2-2.0^**^ | Ref.  1.3 | 1.0-1.8^*^ |
| **Sum of screen based**  >7 hours  ≤7 hours | Ref.  1.2 | 0.9-1.6 | - |  | Ref.  1.0 | 0.7-1.5 | - |  | Ref.  1.1 | 0.9-1.3 | - |  |
| **Sum of non screen based**  >5 hours  ≤5 hours | Ref.  1.3 | 0.9-1.9 | - |  | Ref.  1.6 | 1.2-2.3^**^ | Ref.  1.3 | 0.8-2.0 | Ref.  1.4 | 1.2-1.8^**^ | Ref.  1.2 | 0.9-1.5 |

^a^OR = Odds Ratio, ^b^CI = Confidence Interval; Note: Entry level to the logistic regression model was set at p < 0.10; ^*^ association at p<0.05; ^**^ association at p<0.01; ^***^ association at p<0.001
